# Sargassum Inundations and the Risk of Hypertension Disorders Among Pregnant Women Living in the French Caribbean Island of Martinique

**DOI:** 10.3390/ijerph21121612

**Published:** 2024-12-01

**Authors:** Rishika Banydeen, Mickael Rejaudry Lacavalerie, Loic Savoyen, Alice Monthieux, Mehdi Jean-Laurent, Jonathan Florentin, Fatima Radouani, Hossein Mehdaoui, Dabor Resiere, Remi Neviere

**Affiliations:** 1 Cardiovascular Research Team (UR5_3 PC2E), University of the French West Indies (Université des Antilles), 97200 Fort-de-France, France; rishika.banydeen@chu-martinique.fr (R.B.); mickael.rejaudrylacavalerie@chu-martinique.fr (M.R.L.); fatima.radouanisaint-aime@chu-martinique.fr (F.R.); hossein.mehdaoui@chu-martinique.fr (H.M.); dabor.resiere@chu-martinique.fr (D.R.); 2 Department of Toxicology and Critical Care Medicine, University Hospital of Martinique (CHU Martinique), 97261 Fort-de-France, France; 3 Department of Clinical Physiology, University Hospital of Martinique (CHU Martinique), CS 90632, 97261 Fort-de-France, France; 4 Department of Obstetrics and Gynecology, University Hospital of Martinique (CHU Martinique), 97261 Fort-de-France, France; alice.monthieux@chu-martinique.fr (A.M.);

**Keywords:** Caribbean, sargassum, health risk, hydrogen sulfide (H_2_S), pregnancy, hypertension

## Abstract

Since 2011, Caribbean territories have experienced massive and repeated sargassum seaweed inundations. Once on shore, sargassum degradation through anaerobic metabolism elicits the release of many noxious molecules, including hydrogen sulfide (H_2_S) and ammonia (NH_3_). H_2_S has been long recognized as a malodorous and highly toxic gas, while chronic exposure has not been extensively explored. Our objective was to assess whether pregnant women exposed to sargassum emissions would be more prone to developing hypertensive disorders compared to unexposed women. We conducted a retrospective study including 3020 pregnant women at the Obstetrics Department of the University Hospital of Martinique between 25 January 2016 and 31 July 2020. Exposure was defined as a distance of less than 2 km between the residence/workplace of the women and the sargassum strandings. Multivariate regression retained age, body mass index, sickle cell disease, primipaternity, gestational diabetes and sargassum emissions exposure as independent predictors of hypertensive events in pregnant women. Jointly with previous studies from our group, this study highlights the deleterious effects of sargassum emissions on human health in individuals chronically exposed to low to moderate H_2_S concentrations.

## 1. Introduction

In the last decade, massive sargassum seaweed beaching on the coasts of the Caribbean, Central America, and Brazil has become a real threat, causing major socio-economic, ecological, and health problems [1]. Several hypotheses are possible as to why sargassum has been increasingly washing to shore, including changes in hydrodynamic and wind conditions, increased nutrients from the Amazon due to deforestation and intensification of agriculture in these territories, and global warming of the tropical Atlantic [1,2].

The potential impacts on human health from sargassum influx events include emissions of potentially harmful gases, leaching of heavy metals, and exposure to potentially harmful bacteria and stinging organisms that co-occur with sargassum [1,3,4,5]. Toxic gas exposure typically happens during decomposition, approximately 48 h after sargassum mats wash ashore. During this process, sargassum releases hydrogen sulfide (H_2_S) gas and ammonia (NH_3_), which can cause serious health problems, including neurological, digestive, respiratory, and ophthalmologic symptoms in populations exposed throughout weeks [1,4]. While the health effects of long-term and repeated exposure to this gaseous cocktail are largely unknown, previous studies in geothermal areas have observed that H_2_S exposure might increase morbidity for neurological, respiratory, and cardiovascular diseases [6,7,8]. Geothermal sources have the advantage over other ambient H_2_S-producing entities of not being known to produce other gases with the potential to confound results. Indeed, geothermal gases are mainly carbon dioxide and water vapor, with only small amounts of hydrogen, nitrogen, methane and carbon monoxide [8]. Most of the available knowledge about the health effects of chronic exposure to H_2_S comes from research conducted in Rotorua, New Zealand, which is home to the world’s largest community living over an active geothermal field [8]. In these studies, other possible air pollutants are vehicle emissions, which are limited to the main business street of Rotorua and can be easily identified as a confounding factor, hence allowing to study the health effects of chronic exposure to H_2_S. Of note, sargassum emissions are mainly related to H_2_S production at ranging doses similar to those reported in the Rotorua geothermal field [1,4].

Associations between the hypertensive disorders of pregnancy and gestational air pollution exposure have been previously reported [9,10,11,12,13,14,15]. Epidemiological studies and meta-analyses [11,14] provide strong evidence that various gaseous and particulate pollutants are associated with hypertensive disorders of pregnancy and pre-eclampsia. The impacts of nitrogen oxides (NO_2_, NO_X_) and particulate matter (PM10, PM2.5) on hypertensive disorders of pregnancy were consistently observed [9,10,12,13,15], whereas the implication of ozone (O_3_) exposure remains inconsistent [16]. H_2_S is present in the atmosphere as the result of industrial activities and volcanoes and geothermal vents, as well as being released from wetlands, salt marshes, and estuaries, where it is produced by bacteria during the anaerobic decay of organic sulfur compounds [8,17]. Surprisingly, atmospheric H_2_S has not been previously associated with hypertensive disorders of pregnancy.

Evidence of both the protective role and deleterious effects of H_2_S in cardiovascular diseases, including hypertension, has been demonstrated [18,19,20,21]. Many reports have described the beneficial effects of H_2_S on cardiovascular cellular processes, including the modulation of inflammation, improved cell survival, cytoprotection against oxidative stress, as well as positive effects on mitochondrial metabolic function and biogenesis [18,19,20,21]. As a different concept, cardiovascular H_2_S toxicity has been attributed to the inhibition of cellular enzymes [18,19,20,21,22] and vasoconstriction [23,24,25,26,27,28]. The involvement of H_2_S in the pathophysiology of hypertensive disorders of pregnancy and pre-eclampsia is not clear. It has been reported that mRNA and protein expression of the enzyme cystathionine γ-lyase (CSE) are decreased in the placental tissue of pre-eclamptic women [29]. However, H_2_S plasma levels were found either decreased or increased in women with pre-eclampsia compared to healthy pregnant women [30,31]. 

To date, only a few studies, mainly from our research group, have objectively reported the clinical symptomatology associated with chronic exposure to sargassum gaseous emissions [4,32,33]. In the specific context of human H_2_S exposure to sargassum emissions, we have previously reported that the onset of pre-eclampsia occurred earlier in women living and/or working close coastal sargassum strandings [34]. Whether day-to-day variations in H_2_S levels are associated with increased risk of hypertensive disorders onset during pregnancy has not been previously investigated. The present study aims to analyze the potential relation between environmental exposure to H_2_S gas emitted by decomposing sargassum and pregnancy-related hypertensive disorders. 

## 2. Materials and Methods

### 2.1. Ethical Approval

The present study was conducted in accordance with the amended Declaration of Helsinki (https://www.wma.net/what-we-do/medical-ethics/declaration-of-helsinki/; accessed on 20 September 2024) and Good Clinical Practice guidelines (GCP European Directive 2005/28/EC; accessed on 20 September 2024). Written informed consent was systematically obtained from all patients. The study was approved by the local Institutional Review Board of the University Hospital of Martinique (IRB 2023/033). 

### 2.2. Study Setting 

The French Caribbean Island of Martinique (14.6415° N, 61.0242° W, surface area of 1128 km^2^) is one of the most populated territories in the Caribbean basin (population size of 365,734 in 2023). The east coast of the island is bordered by the Atlantic Ocean, while its west coast faces the Caribbean Sea. Oceanic current and maritime trade winds off the Atlantic coast expose Martinique’s eastern coast to coastal sargassum stranding episodes, i.e., the onshore accumulation and compaction of large amounts of the seaweed. In contrast, the island’s Caribbean shoreline is protected by a mountainous relief, which shields the coastline from sargassum strandings. The sargassum invasion of the Atlantic coastline of Martinique was first noted in 2011, with massive invasions recurring annually or biannually since 2018.

### 2.3. Study Population 

This single-center prospective observational study was conducted at the University Hospital of Martinique from 25 January 2016 to 31 July 2020. Pregnant women, residing in Martinique, were recruited and followed during the study period at the University Hospital’s obstetric center. The study population was restricted to women with pregnancy (determined by ultrasound scan). Other inclusion criteria were a minimal age of 18 years and a gestational age of 20 weeks or more. Exclusion criteria were as follows: inability or refusal to provide informed consent for study participation, pregnancy with more than two babies at a time, and women geographically relocating during the pregnancy period. The latter criterion makes it difficult to determine living/working distance from coastal sargassum stranding sites. 

### 2.4. Study Data

Study data were retrospectively extracted from the administrative and medical databases of the University Hospital of Martinique using the Emergency DX Care software (DXCare 8.2021, Medasys, Dedalus, France). In addition to each subject’s home and work addresses, the data collected retrospectively for study purposes included the patients’ sociodemographic features (age, sex, body mass index, personal and medical history, as well as pregnancy complications and delivery characteristics). 

### 2.5. Definition of Sargassum Exposure (H_2_S)

The patient’s status of exposure to sargassum gaseous emissions was determined based on the residential and work addresses provided. Island residents were considered to be exposed to ambient H_2_S emissions from decomposing sargassum if they lived and/or worked in areas along the Atlantic coast of Martinique, which are known to be impacted by sargassum influxes (Figure S1). 

The levels of exposure were determined on the basis of the measured levels of ambient H_2_S and NH_3_ levels in ambient air, which are gauged using a network of 16 ground sensors. These sensors have been deployed along the Atlantic coast of Martinique since 2016 by Martinique’s air quality observatory (Madininair) and certified by the French Ministry of Ecology, Energy, and Sustainable Development (https://www.madininair.fr/, accessed on 20 September 2024). Ground sensors are located in sensitive areas such as schools, colleges, health establishments, or densely populated areas close to sites of sargassum strandings. Accordingly, pregnant women identified as exposed to increased ambient H_2_S and NH_3_ levels were living and/or working in areas within a distance of <2 km from the nearest ground sensor. For those pregnant women, mean individual H_2_S exposures were approximated by noting the daily concentrations (in ppm) of H_2_S provided by the nearest ground sensor to a patient’s living or working quarters and averaged over the whole pregnancy duration. In contrast, patients living and/or working in the island’s center or along the Caribbean shoreline, in areas located up to 2 km from sargassum stranding sites, were considered to be unexposed to H_2_S originating from decomposing sargassum. This assumption was made on the basis of the virtually nil levels of these gases registered by the mobile sensors deployed in these regions. As such, in light of the lack of sargassum exposure and any other natural, chemical, or industrial source of H_2_S emissions in these areas, populations not residing/working on the Atlantic coast of Martinique were thus considered unexposed.

### 2.6. Definition of Hypertensive Disorders of Pregnancy 

The outcome was hypertensive disorders of pregnancy, which were diagnosed according to the diagnostic criteria of the UK College of Obstetrics and Gynecology [35]. In brief, hypertensive disorders of pregnancy include gestational hypertension, pre-eclampsia (including chronic (pre-existing) hypertension with superimposed pre-eclampsia), and eclampsia. In this study, a history of chronic (pre-existing) hypertension was not considered as related to pregnancy. 

### 2.7. Air Pollution

Air pollution data, collected by Martinique’s air quality observatory, were also analyzed in order to take into account the potential confounding effect of air pollution on pregnancy outcome. Levels of ozone (O_3_), nitrogen dioxide (NO_2_), sulfur dioxide (SO_2_), particulate matter of ≤10 μm in diameter (PM10), and fine particulate matter of ≤2.5 μm in diameter (PM2.5) were considered over the study period. Crude daily data, originating from the different measure stations in Martinique, were obtained for each pollutant (readings provided by Madininair), and monthly aggregated mean concentrations of each pollutant were computed for the whole island. Computed means were then compared to limit values for the protection of human health, set by the French Ministry of Ecology, Energy, and Sustainable Development. Beyond these threshold values for each pollutant, a health risk was considered plausible.

### 2.8. Statistical Analysis

For all descriptive and inferential analyses, the assumption of normal data distribution was analyzed. Mean and 95% confidence intervals were reported for normally distributed variables and median and min-max range for non-normally distributed variables. Categorical variables were presented as absolute values and percentages. The following tests were used for group comparisons: Student’s *t*-test, chi-squared test, and Fisher’s exact test. The level of statistical significance was set at *p* < 0.05. Univariate and multivariate logistic regression models were fitted to assess the independent effect of predictors on pregnancy-related hypertension disorders. Variables with significant association in univariate analysis (*p* < 0.25) were retained for backward stepwise multivariate regression analysis. Associations were quantified using odds ratio (OR) and 95% confidence intervals. All statistical analyses were conducted using the SPSS software 26.0 for Windows (IBM Corp., Armonk, NY, USA).

## 3. Results

**Figure 1:**
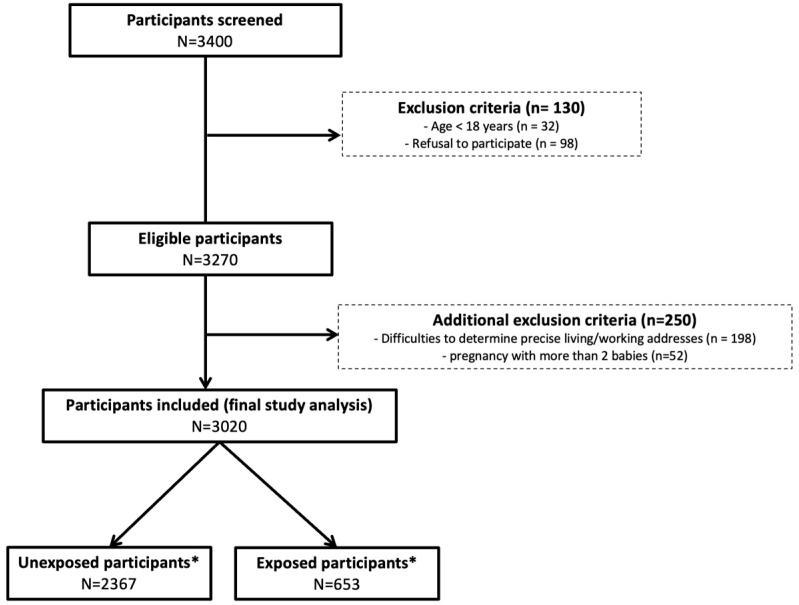
Study flow chart of patient screening and inclusion. * indicates exposure status according to sargassum emissions.

The study population was restricted to pregnant women attending the University Hospital of Martinique during the study period (2016–2020). Overall, 3020 eligible pregnant women with a complete dataset and available home/work addresses were included for analysis. The flow chart of inclusion is displayed in Figure 1. 

The main clinical and biological characteristics of pregnant women according to pregnancy-induced hypertension disorders are presented in Table 1. Compared to pregnant women without hypertension disorders, those with hypertension disorders (11.7%) were older and displayed a higher body weight index. Primiparity and primipaternity were more frequent in pregnant women with hypertension disorders compared to normotensive pregnant women. No significant differences were found for active tobacco use. Medical history of chronic hypertension, type 2 diabetes, sickle cell disease, and personal pre-eclampsia items were more frequently reported in pregnant women with hypertension disorders. Term pregnancy was shorter in pregnant women with hypertension disorders and those women were more prone to complications such as gestational diabetes, threat of premature labor, eclampsia, and HELLP syndrome (Table 1). Compared to pregnant women without hypertension disorders, those with hypertension disorders displayed increased blood markers of liver dysfunction, inflammation, and leukocytosis. Newborn weight and Apgar score were also lower in pregnant women with hypertension disorders. Environmental exposure to active sargassum strandings tends to be more frequent in pregnant women with hypertension disorders but failed to reach statistical significance (*p*-value = 0.073). The mean concentration of H_2_S over the whole pregnancy was found to be higher in women with hypertension disorders compared to normotensive pregnant women (Table 1).

The main characteristics of pregnant women (study population n = 3020) according to sargassum stranding exposure evaluated by the distance between their living/working places and coastline sargassum strandings are presented Table 2. Compared to unexposed pregnant women (n = 2367), those exposed to sargassum strandings (n = 653) were older, while hypertensive disorders and HELLP syndrome were more frequent (Table 2).

On average, the mean H_2_S concentration over the whole period of pregnancy was 0.036 ± 0.236 ppm in exposed subjects. Pregnant women living/working on the Atlantic coast were exposed for 26 days to mean (daily) H_2_S levels of at least 1 ppm. In some areas, patients experienced a maximal number of 12 days of mean (daily) H_2_S concentrations of 5 pm or more. On average, the mean NH_3_ concentration over the whole period of pregnancy was 0.052 ± 0.184 ppm in exposed subjects. None of the pregnant women living/working on the Atlantic coast were exposed to NH_3_ levels up to 8.3 ppm. Of note, H_2_S and NH_3_ recommendations and thresholds from the French High Council for Public Health (Haut Conseil Santé Public (https://www.madininair.fr/, accessed on 20 September 2024) are 1 ppm and 8.3 ppm, respectively.

The main characteristics of pregnant women with hypertension disorders (n = 351) according to the distance between their living/working places and coastline sargassum strandings are presented in Table 3. Pregnant women with hypertension disorders exposed to sargassum emission (living/working places and coastline within 2 km of sargassum strandings) have shorter-term pregnancies, gave birth to a newborn of lower weight, and were more prone to HELLP syndrome complication compared to unexposed pregnant women with hypertension disorders (Table 3). Exposed and unexposed pregnant women with hypertension disorders had similar blood marker levels.

The univariate and multivariate analyses of risk factors for hypertensive disorders in pregnant women are displayed Table 4. The multivariate analysis further highlights this increased risk of hypertension disorders in pregnant women within a living/working distance of 2 km from coastline sargassum stranding sites (odds ratio (OR): 1.59 (1.09–2.34) *p* = 0.017). Significantly increased ORs were also observed for age, BMI, personal history of sickle cell disease, primipaternity, increased weight gain, and gestational diabetes (Table 4). 

The continuous monitoring of air pollutants (O_3_, NOx, NO_2_, and SO_2_) and ambient particulate matter during the study period indicated that these pollutants remained within threshold values defining optimal air quality (recommendations of the French Ministry of Ecology, Energy, and Sustainable Development). The only alert levels observed during the study period concerned ambient particulate matter PM10 concentrations in June 2018 and September 2018, distant time periods from the only massive stranding episode of that year (early March 2018). Because there were no differences in O_3_, NOx, NO_2_, SO_2_, and ambient particulate matters between exposed and unexposed participants, air pollution data were not considered in the logistic univariate and multivariate analyses. 

## 4. Discussion

The present pioneer work, all while confirming the previously reported general clinical syndrome associated with chronic exposure to sargassum emissions [1,3,4], further suggests a potential deleterious effect of these emissions on cardiovascular function in pregnant women. For the first time, we described that chronic exposure to sargassum H_2_S is potentially associated with the risk of hypertension disorders during pregnancy. In pregnant women, sargassum emission exposure was found an independent risk factor of hypertensive disorders in addition to the typical risk factors of hypertensive disorders of pregnancy such as age, body mass index, primipaternity, and gestational diabetes [35]. 

The accumulation of sargassum has been increasingly causing environmental and socio-economic challenges in recent years, particularly along the Caribbean coasts [1,2,3,4]. Despite active collecting, the inundation and compaction of large amounts of sargassum seaweed on shore result in their bacterial putrefaction, leading to the production of nonvolatile and volatile compounds, including hydrogen sulfide (H_2_S) and ammonia (NH_3_). While the acute toxicities of H_2_S and NH_3_ have been well established, the clinical symptomatology associated with chronic exposure to sargassum gaseous emissions have not been extensively studied. 

In our study, exposure to sargassum emissions was indirectly determined by the distance between their residence and/or workplace and sargassum strandings. Pregnant women identified as exposed to increased ambient H_2_S and NH_3_ levels were living and/or working in areas within a distance of <2 km from the nearest ground sensor. H_2_S exposure was based on specific sensors deployed since 2016 along the Atlantic coast in anticipation of massive deposits. It was estimated in our study that pregnant women living/working in areas ≤2 km from sargassum stranding sites were exposed to a mean H_2_S concentration of 0.17 ± 0.49 ppm during their overall pregnancy duration. 

The observation of a potential higher risk of hypertensive disorders in pregnant women living/working near sargassum strandings is in contradiction with the large body of literature, which rather describes H_2_S as a vasodilation molecule with protective action in systemic arterial hypertension [18,19,20,21]. At the vascular level, H_2_S can induce endothelium-independent and endothelium-dependent vasorelaxation through mechanisms involving the activation of K_ATP_ channels on vascular smooth muscle cells, voltage-dependent calcium channel inactivation, and release of the endothelium-derived hyperpolarizing factor [21,22]. On the contrary, H_2_S can induce vasoconstriction under certain conditions through mechanisms involving the quenching or inactivation of nitric oxide (NO) and the inhibition of endothelial cell NO synthase. NO-independent mechanisms have also been reported, including changes in calcium and cyclic adenosine monophosphate (cAMP) concentrations in vascular smooth muscle cells and Rho kinase signaling pathway activation [20,23,24,25,26,27,36,37]. Furthermore, it was recently shown that the single electron oxidation of H_2_S by oxyhemoglobin generates a hydrosulfide radical (HS^•^), which causes vasoconstriction via an L-type calcium channel-dependent pathway and thus can exert systemic arterial hypertension [28]. Therefore, the cardiovascular effects of H_2_S are by nature bidirectional. 

In addition to the vascular dysfunction elicited by H_2_S, previous experimental studies have reported that inhaling H_2_S may induce central metabolic and hemodynamic changes, including reduced energy expenditure and hypothermia, and reduced cardiac output, while blood pressure and stroke volume remained unaffected [38,39]. It is, hence, possible that H_2_S may have limited the physiological increase in cardiac output during the course of pregnancy, which may induce an excessive vasoconstriction and increased blood pressure in exposed pregnant women. Such central hemodynamic maladaptation along with the direct vascular effects of H_2_S could be responsible for the pregnancy hypertensive disorders observed in our study. In our study, standard biochemistry markers, C reactive protein, blood count, and coagulation tests did not show significant differences between pregnant women exposed to sargassum emissions and those without. Due to the retrospective nature of our study, no attempt was made to explore cardiovascular function through cardiac biomarkers such brain natriuretic peptide (BNP) and N-terminal proBNP (NT-proBNP) and vascular NO biodisponibility through the blood concentration of nitrites/nitrates (NO_2_/NO_3_).

Studies seeking to analyze adverse human health effects associated with chronic exposure to H_2_S have defined low-level exposures as H_2_S concentrations below 0.1 ppm, medium-level ones as between 0.1 and 1 ppm, and high-level exposures as above 1 ppm [8]. Ambient low H_2_S concentration may be related to a variety of sources including geothermal and volcanic emissions and anthropogenic sources, such as confined animal feeding operations, oil and gas facilities, paper mills, and waste-water plants. At low H_2_S concentrations, chronic exposure has been associated with either weak positive and negative associations with outcomes such as ischemic heart disease mortality, acute myocardial infarction mortality, and hospitalization for cardiovascular diseases. Pregnant women living/working in areas ≤2 km from sargassum stranding sites who developed hypertensive disorders were exposed to a mean H_2_S concentration of 0.17 ± 0.49 ppm, which may be considered a medium level of H_2_S exposure. The deleterious cardiovascular effects of sargassum emissions in our study are consistent with previous reports showing that exposure to low-medium H_2_S levels was associated with increased incidence of cardiovascular diseases, including hypertension and cardiovascular risk factors [40,41,42,43,44].

## 5. Study Limitations

Several study limitations are to be noted in our exploratory research. Firstly, exposure to sargassum stranding was approximated by the closest ground H_2_S sensor, which might not reflect actual individual exposure. Unfortunately, a more accurate assessment of individual exposure was not possible with the current study design, notably by the implementation of personal gas detectors or the consideration of a proxy exposure indicator better modelling gas dispersion effects according to distance, landscape, and climatic parameters. Also, the sensor detection of gases emitted by decomposing sargassum seaweed was solely limited to ambient H_2_S and NH_3_, which might not reflect the overall patient exposure to the multitude of toxic gases contained in these emissions, as well as any potential physicochemical interaction between the different gases present. Of note, previous studies have detected heavy metals in sargassum (arsenic (As) and cadmium (Cd)) as well as other potentially toxic elements (lithium (Li), molybdenum (Mo), cesium (Cs), and uranium (U)) that may impact cardiovascular health. Specifically, arsenic exposure has been associated with hypertensive disorders of pregnancy. It is thus expected that, since arsenic is released by sargassum decomposition, chronic arsenic exposure could play a part in an adverse hypertensive outcome in pregnant women.

Secondly, while remaining under threshold values defining optimal air quality, the association between air pollution (O_3_, NOx, NO_2_, SO_2_, and fine particle matter PM10 and PM2.5) and hypertensive disorders was not specifically evaluated. Numerous studies have, however, identified investigated the impact of air pollution on human health, which strongly suggest that exposure to fine particles and air pollutant gases is associated with the atherosclerosis process and onset of hypertensive disorders of pregnancy. Sargassum influxes can accompanied by specific climatic and air pollution episodes such as Saharan dust haze. During the study period of January 2016–July 2020, the only alert for Saharan dust haze was recorded in June 2018 and in September 2018, a few months after the mass stranding described that year. 

Thirdly, the retrospective nature of our study was responsible for the absence of consistent information (medical history) available in the medical patient file to adjust with pre-existing chronic hypertension. We acknowledge that individuals with pre-existing hypertension were at higher risks of developing gestational hypertension and pre-eclampsia during pregnancy. Also, whether women were exposed or not to sargassum emission before pregnancy was not evaluated. The lack of precise residential data did not allow to study the association with a specific sensor data, hence individual concentrations of H_2_S and NH_3_.

Fourthly, confounding factors such as those related to sociodemographic and economic levels, housing, or maternal education were not considered. This is attributed to the retrospective nature of our study and the resulting lack of data reliability. In our study, we acknowledge that 49% of the patient’s report having employment, with 90% working near their residence. 

## 6. Conclusions

Our work highlights a potential association between exposure to sargassum emissions and the risk of hypertensive events in pregnant women. The present study results strongly support the deleterious effect on cardiovascular health of pregnant women chronically exposed to sargassum gas emissions. These preliminary findings emphasize the importance of implementing health measures by health authorities to prevent pregnant women from being in areas where sargassum strands ashore.

## Figures and Tables

**Table 1:** Main clinical and biological characteristics of pregnant women (study population N = 3020) according to hypertensive disorders of pregnancy.

Characteristics		Hypertensive Disorders of Pregnancy		
	All N = 3020	Yes N = 351	No N = 2669	*p*-Value
Age, years	29.8 ± 6.2	31.2 ± 7.0	29.6 ± 6.1	<0.001 *
Age > 30 years	1431 (47.4%)	197 (56.1%)	1234 (46.2%)	<0.001 *
BMI, kg/m^2^	26.3 ± 6.8	30.1 ± 8.0	25.8 ± 6.4	<0.001 *
BMI > 30 kg/m^2 n = 2897^	1325 (43.9%)	224 (63.8%)	1101 (41.3%)	<0.001 *
Primiparity	1430 (47.4%)	188 (53.6%)	1247 (46.7%)	0.001 *
Primipaternity ^n = 2050^	1675 (82%)	215 (88%)	1460 (81%)	0.004 *
Twin pregnancy ^n = 3018^	71 (2.4%)	10 (2.8%)	61 (2.3%)	0.308
Active tobacco use ^n = 3014^	196 (6.5)	24 (6.9%)	172 (6.5%)	0.417
Medical history				
Endometriosis	60 (2.0%)	7 (2.0%)	53 (2.0%)	0.557
Thyroid diseases	55 (1.8%)	4 (1.1%)	51 (1.9%)	0.216
Chronic hypertension ^n = 3016^	87 (2.9%)	54 (15.5%)	33 (1.2%)	<0.001 *
Diabetes	90 (3.0%)	26 (7.4%)	64 (2.4%)	<0.001 *
Sickle cell disease	10 (0.3%)	4 (1.1%)	6 (0.2%)	0.203
Polycystic ovary syndrome	49 (1.6%)	8 (2.3%)	41 (1.5%)	0.365
Personal pre-eclampsia	56 (1.9%)	25 (7.1%)	31 (1.2%)	<0.001 *
Pregnancy outcome				
Term pregnancy, weeks	38.7 ± 3.0	37.6 ± 3.9	38.9 ± 2.8	<0.001 *
Weight gain, kg	11.0 ± 6.0	12.0 ± 7.4	10.9 ± 5.8	0.010 *
Newborn weight, kg	3.04 ± 0.67	2.77 ± 0.89	3.07 ± 0.62	<0.001 *
Apgar score 1th min	8.9 ± 2.5	8.0 ± 3.2	9.0 ± 2.3	<0.001 *
Apgar score 5th min	9.5 ± 1.7	8.9 ± 2.5	9.6 ± 1.6	<0.001 *
Umbilical cord blood lactate	4.4 ± 3.4	5.2 ± 4.8	4.3 ± 3.0	0.061
Pregnancy complication				
Gestational diabetes	274 (9.1%)	70 (20.0%)	204 (7.7%)	<0.001 *
Threat of premature labor	690 (22.8%)	111 (31.6%)	579 (21.7)	<0.001 *
Pre-eclampsia	194 (6.4%)	194 (55.3%)	0 (0%)	<0.001 *
Eclampsia	4 (0.1%)	4 (1.1%)	0 (0.0)	<0.001 *
HELLP syndrome	26 (0.9%)	26 (7.4%)	0 (0)	<0.001 *
Obstetrical hemorrhage	127 (4.2%)	28 (8.0%)	99 (3.7%)	<0.001 *
Neonatal death	66 (2.2%)	19 (5.4%)	47 (1.8%)	<0.001 *
Biochemistry markers				
Urea, mmol/L	3.0 ± 1.3	3.1 ± 1.1	3.0 ± 1.3	0,979
Creatinine, µmol/L	58.4 ± 13.5	58.1 ± 11.4	58.5 ± 13.8	0.833
Total bilirubin, µmol/L	9.7 ± 12.3	8.8 ± 6.6	10.1 ± 14.3	0.171
Aspartate aminotransferase, IU/L	43 ± 105	53 ± 111	38 ± 101	0.046 *
Alanine aminotransferase, IU/L	30 ± 77	39 ± 85	25 ± 71	0.008 *
Alkaline phosphatase, IU/L	181 ± 159	201 ± 205	171 ± 130	0.009 *
C reactive protein, mg/L	29 ± 47	38 ± 55	28 ± 46	0.003 *
Blood count and coagulation tests				
Red blood cells, million/mm^3^	4.0 ± 0.5	4.0 ± 0.6	4.0 ± 0.5	0.514
Hemoglobin, g/dL	11.0 ± 1.4	11.0 ± 1.5	11.0 ± 1.4	0.521
Hematocrit, %	33.9 ± 3.9	33.7 ± 4.5	33.9 ± 3.9	0.251
White blood cells, mm^−3^	11.9 ± 4.3	12.9 ± 4.9	11.7 ± 4.2	<0.001 *
Neutrophils, %	73.8 ± 10.0	76.5 ± 9.2	73.3 ± 10.0	<0.001 *
Lymphocytes, %	18.1 ± 8.4	16.4 ± 7.7	18.4 ± 8.5	<0.001 *
Monocytes, %	7.7 ± 2.3	7.3 ± 2.4	7.8 ± 2.3	0.001 *
Eosinophils, %	1.7 ± 1.7	2.1 ± 1.6	1.6 ± 1.7	<0.001 *
Basophils, %	0.37± 0.21	0.43 ± 0.23	0.36 ± 0.20	<0.001 *
Platelets, ×10 µL^−1^	232 ± 70	221 ± 73	234 ± 69	0.001 *
Fibrinogen, g/L	4.7 ± 1.0	4.6 ± 1.1	4.7 ± 1.0	0.031 *
PT, %	104 ± 14	103 ± 15	104 ± 14	0.111
APTT, s.	29 ± 9	29 ± 3	28 ± 10	0.375
Environmental exposure				
H_2_S concentration (ppm)	0.036 ± 0.236	0.073 ± 0.375	0.032 ± 0.211	0.002 *
NH_3_ concentration (ppm)	0.052 ± 0.184	0.063 ± 0.207	0.037 ± 0.146	0.188
Active sargassum strandings	653 (21.6%)	87 (24.8%)	566 (21.2)	0.073

Abbreviations: BMI, body mass index; HELLP, Hemolysis, elevated liver enzymes, and low platelet count; TP, prothrombin time; APTT, activated partial thrombosplatin time. Data are represented as mean ± SD or absolute value (%); * statistical significance set at *p* < 0.05.

**Table 2:** Main clinical and biological characteristics of pregnant women (study population N = 3020) according to sargassum exposure.

Characteristics		Sargassum Stranding Exposure		
	All N = 3020	Yes (N = 653)	No N = 2367	*p*-Value
Age, years	29.8 ± 6.2	30.3 ± 6.6	29.7 ± 6.1	0.021 *
Age > 30 years	1431 (47.4%)	322 (49.3%)	1109 (46.9%)	0.142
BMI, kg/m^2^	26.3 ± 6.8	26.2 ± 7.5	26.2 ± 6.6	0.241
BMI > 30 kg/m^2 n = 2897^	1325 (43.9%)	320 (49%)	1110 (47%)	0.386
Primiparity	1430 (47.4%)	188 (54%)	1247 (47%)	0.607
Primipaternity ^n = 2050^	1675 (82%)	371 (82%)	1304 (82%)	0.394
Twin pregnancy ^n = 3018^	71 (2.4%)	12 (1.8%)	59 (2.5%)	0.204
Active tobacco use ^n = 3014^	196 (6.5)	39 (6.0%)	157 (6.6%)	0.309
Medical history				
Endometriosis	60 (2.0%)	18 (2.8%)	42 (1.8%)	0.079
Thyroid diseases	55 (1.8%)	13 (2.0%)	42 (1.8%)	0.409
Chronic hypertension ^n = 3016^	87 (2.9%)	23 (3.5%)	64 (2.7%)	0.164
Diabetes	90 (3.0%)	24 (3.7%)	66 (2.8%)	0.147
Sickle cell disease	10 (0.3%)	2 (0.3%)	8 (0.3%)	0.628
Polycystic ovary syndrome	49 (1.6%)	8 (1.2%)	41 (1.7%)	0.237
Personal pre-eclampsia	56 (1.9%)	16 (2.5%)	40 (1.7%)	0.134
Pregnancy outcome				
Term pregnancy, weeks	38.7 ± 3.0	38.6 ± 3.0	38.8 ± 3.0	0.364
Weight gain, kg	11.0 ± 6.0	11.3 ± 5.7	11.0 ± 6.1	0.322
Newborn weight, kg	3.04 ± 0.67	3.01 ± 0.67	3.04 ± 0.66	0.237
Apgar score 1th min	8.9 ± 2.5	8.8 ± 2.5	8.9 ± 2.5	0.235
Apgar score 5th min	9.5 ± 1.7	9.5 ± 1.7	9.5 ± 1.7	0.339
Umbilical cord blood lactate	4.4 ± 3.4	4.5 ± 3.0	4.4 ± 3.5	0.800
Pregnancy complication				
Gestational diabetes	274 (9.1%)	53 (8.1%)	221 (9.4%)	0.186
Threat of premature labor	690 (22.8%)	150 (23.0%)	540 (22.8)	0.485
Hypertensive disorders	353 (11.7%)	89 (13.6%)	264 (11.2%)	0.049 *
Pre-eclampsia	194 (6.4%)	49 (7.5%)	145 (6.1%)	0.120
Eclampsia	4 (0.1%)	0 (0%)	4 (0.2%)	0.377
HELLP syndrome	26 (0.9%)	11 (1.7%)	15 (0.6%)	0.014 *
Obstetrical hemorrhage	127 (4.2%)	28 (4.3%)	99 (4.2%)	0.489
Neonatal death	66 (2.2%)	13 (2.0%)	53 (2.2%)	0.908
Biochemistry markers				
Urea, mmol/L	3.0 ± 1.3	3.1 ± 1.1	3.0 ± 1.3	0.828
Creatinine, µmol/L	58.4 ± 13.5	58.1 ± 11.4	58.5 ± 13.8	0.762
Total bilirubin, µmol/L	9.7 ± 12.3	8.8 ± 6.6	10.1 ± 14.3	0.434
Aspartate aminotransferase, IU/L	43 ± 105	53 ± 111	38 ± 101	0.144
Alanine aminotransferase, IU/L	30 ± 77	39 ± 85	25 ± 71	0.180
Alkaline phosphatase, IU/L	181 ± 159	201 ± 205	171 ± 130	0.314
C reactive protein, mg/L	29 ± 47	29 ± 44	30 ± 48	0.734
Blood count and coagulation tests				
Red blood cells, million/mm^3^	4.0 ± 0.5	4.0 ± 0.6	4.0 ± 0.5	0.466
Hemoglobin, g/dL	11.0 ± 1.4	10.9 ± 1.4	11.0 ± 1.4	0.628
Hematocrit, %	33.9 ± 3.9	33.8 ± 4.0	33.9 ± 3.9	0.655
White blood cells, mm^−3^	11.9 ± 4.3	11.7 ± 4.0	11.9 ± 4.4	0.319
Neutrophils, %	73.8 ± 10.0	73.7 ± 9.3	73.8 ± 10.0	0.866
Lymphocytes, %	18.1 ± 8.4	16.4 ± 7.7	18.4 ± 8.5	0.920
Monocytes, %	7.7 ± 2.3	7.7 ± 2.2	7.7 ± 2.3	0.475
Eosinophils, %	1.7 ± 1.7	1.9 ± 1.8	1.7 ± 1.6	0.023 *
Basophils, %	0.37± 0.21	0.38 ± 0.21	0.37 ± 0.21	0.366
Platelets, ×10 µL^−1^	232 ± 70	234 ± 76	231 ± 68	0.362
Fibrinogen, g/L	4.7 ± 1.0	4.6 ± 1.1	4.7 ± 1.0	0.946
PT, %	104 ± 14	103 ± 15	104 ± 14	0.282
APTT, s.	29 ± 9	29 ± 7	29 ± 10	0.916
Environmental exposure				
H_2_S concentration (ppm)	0.036 ± 0.236	0.169 ± 0.486	0 ± 0	<0.001 *
NH_3_ concentration (ppm)	0.052 ± 0.184	0.073 ± 0.242	0 ± 0	<0.001 *
Mean NO_2_ concentration, µg·m^−3^	7.2 ± 0.9	7.2 ± 0.9	7.3 ± 0.9	0.123
Mean SO_2_ concentration, µg·m^−3^	7.7 ± 3.8	7.5 ± 3.9	7.8 ± 3.7	0.496
Mean O_3_ concentration, µg·m^−3^	45.0 ± 8.4	44.7 ± 8.4	45.2 ± 8.5	0.634
Mean PM10 concentration, µg·m^−3^	22.5 ± 3.2	22.6 ± 3.3	22.4 ± 3.1	0.598
Mean PM2.5 concentration, µg·m^−3^	12.8 ± 3.1	13.2 ± 2.9	12.3 ± 4.1	0.673

Abbreviations: APTT, activated partial thrombosplatin time; BMI, body mass index; HELLP, Hemolysis, elevated liver enzymes, andlow platelet count; NO_2_, nitrogen dioxide; O_3_, ozone; PM2.5, fine particulate matter of ≤2.5 μm in diameter; PM10; particulate matter of ≤10 μm in diameter; SO_2_, sulfur dioxide; TP, prothrombin time. Data are represented as mean ± SD or absolute value (%); * statistical significance set at *p* < 0.05.

**Table 3:** Main clinical and biological characteristics of pregnant women with hypertensive disorders (study population (N = 351) according to according to sargassum exposure.

Characteristics		Sargassum Stranding Exposure		
	All N = 351	Yes N = 87	No N = 264	*p*-Value
Age, years	31.2 ± 7.0	31.6 ± 7.3	31.1 ± 6.9	0.523
Age > 30 years	197 (56.1%)	50 (56.2%)	147 (55.7%)	0.518
BMI, kg/m^2^	30.1 ± 8.0	29.6 ± 7.7	30.3 ± 8.1	0.481
BMI > 30 kg/m^2^	224 (63.8%)	53 (63.1%)	171 (66.3%)	0.342
Primiparity	188 (53.6%)	48 (55%)	140 (53%)	0.129
Primipaternity ^n = 245^	215 (88%)	57 (89%)	158 (87%)	0.451
Twin pregnancy	10 (2.8%)	1 (1.1%)	9 (3.4%)	0.245
Active tobacco use ^n = 349^	24 (6.9%)	8 (9.3%)	16 (6.1%)	0.214
Medical history				
Endometriosis	7 (2.0%)	4 (4.6%)	3 (1.1%)	0.067
Thyroid diseases	4 (1.1%)	1 (1.1%)	3 (1.1%)	0.682
Chronic hypertension	54 (15.4%)	18 (20.7%)	36 (13.6%)	0.082
Diabetes	26 (7.4%)	5 (5.7%)	21 (8.0%)	0.339
Sickle cell disease	4 (1.1%)	0 (0%)	4 (1.5%)	0.318
Polycystic ovary syndrome	8 (2.3%)	1 (1.1%)	7 (2.7%)	0.370
Personal pre-eclampsia	25 (7.1%)	10 (11.5%)	15 (5.7%)	0.061
Pregnancy outcome				
Term pregnancy, weeks	37.6 ± 3.9	37.6 ± 3.9	38.9 ± 2.8	0.006 *
Weight gain, kg	12.0 ± 7.4	11.7 ± 7.2	12.0 ± 7.5	0.714
Newborn weight, kg	2.77 ± 0.89	2.56 ± 0.96	2.84 ± 0.85	0.011 *
Apgar score 1th min	8.0 ± 3.2	7.5 ± 3.5	8.1 ± 3.2	0.143
Apgar score 5th min	8.9 ± 2.5	8.5 ± 3.0	9.1 ± 2.4	0.076
Umbilical cord blood lactate	5.2 ± 4.8	4.2 ± 3.0	5.6 ± 5.3	0.399
Pregnancy complication				
Gestational diabetes	70 (20.0%)	17 (19.8%)	53 (20.1%)	0.543
Threat of premature labor	111 (31.6%)	31 (35.6%)	80 (30.3%)	0.213
Pre-eclampsia	194 (55.3%)	49 (56.3%)	145 (54.9%)	0.460
Eclampsia	4 (1.1%)	0 (0%)	4 (1.5)	0.318
HELLP syndrome	26 (7.4%)	11 (12.6%)	15 (5.7%)	0.032 *
Obstetrical hemorrhage	28 (8.0%)	8 (9.3%)	20 (7.6%)	0.377
Neonatal death	19 (5.4%)	13 (2.0%)	6 (2.2%)	0.198
Biochemistry markers				
Urea, mmol/L	3.1 ± 1.1	2.8 ± 1.1	3.2 ± 1.1	0,248
Creatinine, µmol/L	58.1 ± 11.4	56.1 ± 14.9	59.1 ± 10.2	0.419
Total bilirubin, µmol/L	8.8 ± 6.6	8.7 ± 7.3	9.0 ± 6.4	0.171
Aspartate aminotransferase, IU/L	53 ± 111	71 ± 181	46 ± 67	0.082
Alanine aminotransferase, IU/L	39 ± 85	51 ± 120	35 ± 67	0.149
Alkaline phosphatase, IU/L	201 ± 205	238 ± 365	188 ± 80	0.062
C reactive protein, mg/L	38 ± 55	40 ± 58	36 ± 53	0.632
Blood count and coagulation tests				
Red blood cells, million/mm^3^	4.0 ± 0.6	3.9 ± 0.6	4.0 ± 0.6	0.100
Hemoglobin, g/dL	11.0 ± 1.5	10.8 ± 1.5	11.0 ± 1.5	0.415
Hematocrit, %	33.7 ± 4.5	33.7 ± 4.5	33.9 ± 3.9	0.251
White blood cells, mm^−3^	12.9 ± 4.9	12.8 ± 4.3	12.9 ± 5.1	0.841
Neutrophils, %	76.5 ± 9.2	76.2 ± 9.1	76.6 ± 9.3	0.709
Lymphocytes, %	16.4 ± 7.7	17.0 ± 7.8	16.2 ± 7.6	0.412
Monocytes, %	7.3 ± 2.4	7.5 ± 2.5	7.3 ± 2.4	0.377
Eosinophils, %	2.1 ± 1.6	2.2 ± 1.7	2.0 ± 1.6	0.472
Basophils, %	0.43± 0.23	0.46 ± 0.25	0.42 ± 0.22	0.201
Platelets, ×10 µL^−1^	221 ± 73	224 ± 91	218 ± 67	0.556
Fibrinogen, g/L	4.6 ± 1.1	4.6 ± 1.2	4.5 ± 1.1	0.547
PT, %	103 ± 15	102 ± 15	103 ± 14	0.546
APTT, s.	29 ± 3	29 ± 3	29 ± 3	0.754
Environmental exposure				
H_2_S concentration (ppm)	0.073 ± 0.375	0.297 ± 0.713	0 ± 0	<0.001 *
NH_3_ concentration (ppm)	0.047 ± 0.181	0.0797 ± 0.236	0 ± 0	<0.001 *
Mean NO_2_ concentration, µg·m^−3^	7.2 ± 0.9	7.2 ± 0.9	7.3 ± 0.9	0.123
Mean SO_2_ concentration, µg·m^−3^	7.7 ± 3.8	7.5 ± 3.9	7.8 ± 3.7	0.496
Mean O_3_ concentration, µg·m^−3^	45.0 ± 8.4	44.7 ± 8.4	45.2 ± 8.5	0.634
Mean PM10 concentration, µg·m^−3^	22.5 ± 3.2	22.6 ± 3.3	22.4 ± 3.1	0.598
Mean PM2.5 concentration, µg·m^−3^	13.4 ± 2.3	13.0 ± 3.1	13.8 ± 2.4	0.676

Abbreviations: APTT, activated partial thrombosplatin time; BMI, body mass index; HELLP, Hemolysis, elevated liver enzymes, and low platelet count; NO_2_, nitrogen dioxide; O_3_, ozone; PM2.5, fine particulate matter of ≤2.5 μm in diameter; PM10; particulate matter of ≤10 μm in diameter; SO_2_, sulfur dioxide; TP, prothrombin time. Data are represented as mean ± SD or absolute value (%); * statistical significance set at *p* < 0.05.

**Table 4:** Logistic univariate and multivariate analyses of risk factors for hypertensive disorders in pregnant women.

	Univariate Analysis		Multivariate Analysis	
	OR (95% CI)	*p*-Value	OR (95% CI)	*p*-Value
Age	1.045 (0.997–1.09)	0.159 *	1.05 (1.02–1.07)	0.001
BMI	1.07 (1.03–1.10)	<0.001 *	1.08 (1.05–1.10)	<0.001
Nulliparity	0.91 (0.75–1.10)	0.339		
Active tobacco use	1.29 (0.67–2.47)	0.448		
Medical history				
Endometriosis	0.72 (0.24–2.18)	0.562		
Diabetes	2.09 (0.87–5.03)	0.099 *		
Sickle cell disease	18.64 (1.95–178.30)	0.011 *	18.11 (2.15–152.57)	0.008
Polycystic ovary syndrome	2.06 (0.83–5.14)	0.120 *		
Pregnancy				
Primipaternity	2.37 (1.30–4.33)	0.005 *	2.52 (1.50–4.24)	0.001
Twin pregnancy	0.83 (0.28–2.42)	0.729		
Weight gain	1.06 (1.03–1.08)	<0.001 *	1.05 (1.03–1.08)	<0.001
Gestational diabetes	1.82 (1.08–3.07)	0.024 *	2.04 (1.26–3.30)	0.004
Environmental exposure				
Sargassum stranding (H_2_S exposure)	1.37 (0.90–2.10)	0.146 *	1.59 (1.09–2.34)	0.017

Abbreviations: BMI, body mass index; OR: odds ratio; CI: Confidence Interval. Variables with a significant association in univariate analysis (* *p* < 0.25) were considered for multivariate analysis. Variables entered into the multivariate modeling: age, BMI, primipaternity, history of diabetes, history of sickle cell disease, weight gain, gestational diabetes, and sargassum stranding exposure. Statistical significance level was set at *p* < 0.05.

## Data Availability

The data that support the findings of this study are available from Pr. NEVIERE Remi from the Department of Clinical Physiology (CHU Martinique). Restrictions, however, apply to the availability of these data, which were used under license for the current study, and hence, are not publicly available. Study data will be available from the authors upon reasonable request and with permission from NEVIERE Remi (remi.neviere@chu-martinique.fr).

## Supplementary Materials

The following supporting information can be downloaded at: https://www.mdpi.com/article/10.3390/ijerph21121612/s1, Figure S1: Schematic map of the Martinique Island (black circles indicate the name of the city). Sargassum stranding intensity is color-coded (green: moderate intensity; orange: high intensity; red: very high intensity). Surface of the circle indicates the number of people exposed to emissions produced by decomposing sargassum in 2018.
