# Supplementary material for: Sargassum Inundations and the Risk of Hypertension Disorders Among Pregnant Women Living in the French Caribbean Island of Martinique
Source: Int J Environ Res Public Health. 2024 Dec 1;21(12):1612. doi: 10.3390/ijerph21121612 (PMC11675253; doi:10.3390/ijerph21121612)
Supplement: Supplementary file 1 [file ijerph-21-01612-s001.zip › ijerph-3265081-supplementary.pdf]

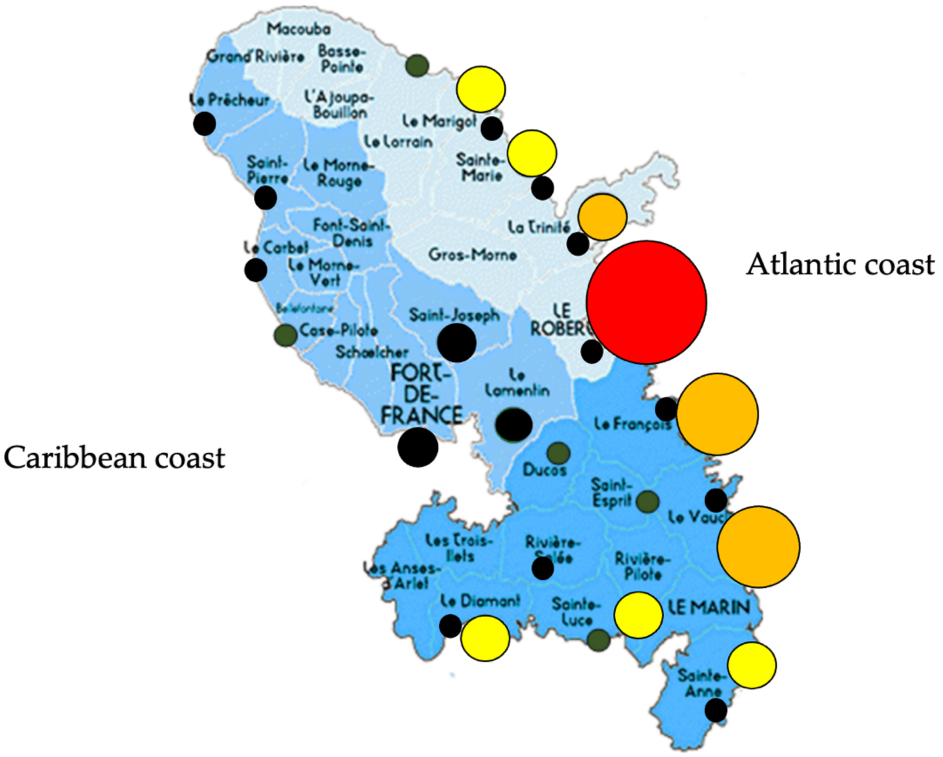

Supplemental Figure S1: Schematic map of the Martinique Island (black circles indicate name of the city. Sargassum stranding intensity is color code (green: moderate intensity, orange: high intensity, red: very high intensity). Surface of the circle indicates the number of people exposed to emission produced by decomposing sargassum in 2018.
